# Loss of Tumor Suppressor CYLD Expression Triggers Cisplatin Resistance in Oral Squamous Cell Carcinoma

**DOI:** 10.3390/ijms20205194

**Published:** 2019-10-20

**Authors:** Naoki Suenaga, Mimi Kuramitsu, Kanae Komure, Ayumi Kanemaru, Kanako Takano, Kazuya Ozeki, Yuka Nishimura, Ryoji Yoshida, Hideki Nakayama, Satoru Shinriki, Hideyuki Saito, Hirofumi Jono

**Affiliations:** 1 Department of Clinical Pharmaceutical Sciences, Graduate School of Pharmaceutical Sciences, Kumamoto University, Kumamoto 860-8556, Japan; 167y3104@st.kumamoto-u.ac.jp (N.S.); mimiku826@gmail.com (M.K.); komu4944@yahoo.co.jp (K.K.); 197y2002@st.kumamoto-u.ac.jp (A.K.); kmn.fbz6@gmail.com (K.T.); 149p1010@st.kumamoto-u.ac.jp (K.O.); 155p1036@st.kumamoto-u.ac.jp (Y.N.); saitohide@kuh.kumamoto-u.ac.jp (H.S.); 2 Department of Oral and Maxillofacial Surgery, Faculty of Life Sciences, Kumamoto University, Kumamoto 860-8556, Japan; ryoshida1126@gmail.com (R.Y.); hinakaya@kumamoto-u.ac.jp (H.N.); 3 Department of Molecular Laboratory Medicine, Graduate School of Medical Sciences, Kumamoto University, Kumamoto 860-8556, Japan; satorus@kuh.kumamoto-u.ac.jp; 4 Department of Pharmacy, Kumamoto University Hospital, Kumamoto 860-8556, Japan

**Keywords:** cylindromatosis, nuclear factor-κB, cisplatin resistance, oral squamous cell carcinoma

## Abstract

Cisplatin is one of the most effective chemotherapeutic agents commonly used for several malignancies including oral squamous cell carcinoma (OSCC). Although cisplatin resistance is a major obstacle to effective treatment and is associated with poor prognosis of OSCC patients, the molecular mechanisms by which it develops are largely unknown. Cylindromatosis (CYLD), a deubiquitinating enzyme, acts as a tumor suppressor in several malignancies. Our previous studies have shown that loss of CYLD expression in OSCC tissues is significantly associated with poor prognosis of OSCC patients. Here, we focused on CYLD expression in OSCC cells and determined whether loss of CYLD expression is involved in cisplatin resistance in OSCC and elucidated its molecular mechanism. In this study, to assess the effect of CYLD down-regulation on cisplatin resistance in human OSCC cell lines (SAS), we knocked-down the CYLD expression by using CYLD-specific siRNA. In cisplatin treatment, cell survival rates in CYLD knockdown SAS cells were significantly increased, indicating that CYLD down-regulation caused cisplatin resistance to SAS cells. Our results suggested that cisplatin resistance caused by CYLD down-regulation was associated with the mechanism through which both the reduction of intracellular cisplatin accumulation and the suppression of cisplatin-induced apoptosis via the NF-κB hyperactivation. Moreover, the combination of cisplatin and bortezomib treatment exhibited significant anti-tumor effects on cisplatin resistance caused by CYLD down-regulation in SAS cells. These findings suggest the possibility that loss of CYLD expression may cause cisplatin resistance in OSCC patients through NF-κB hyperactivation and may be associated with poor prognosis in OSCC patients.

## 1. Introduction

Oral cancer is one of the most common types of human cancer worldwide [1]. Oral cancer includes 40% in head and neck cancer, and more than 90% is oral squamous cell carcinoma (OSCC) histologically [2]. Despite advances in early detection, diagnosis, and multimodal treatment, the 5-year survival rate for patients with OSCC has not changed appreciably for the past 30 years [3]. In fact, surgical therapy often has significant effects on swallowing, speech, and physical appearance. Chemotherapy in addition to radiation treatment has shown efficacy in organ preservation in some sites in the head and neck but has resulted in limited improvement in survival rates. Therefore, appropriate treatment of OSCC remains one of the most difficult challenges, and especially development of minimally invasive non-surgical treatments is urgently required.

Although chemotherapy in OSCC is a useful treatment for patients with advanced cancers and makes tumors smaller, preventing recurrence and metastasis and improving prognosis, development of tumor drug resistance is responsible for poor overall survival of patients with most types of cancer [4]. Cisplatin (cis-diamminedichloroplatinum II), an alkylating agent, is one of the most effective chemotherapeutic agents commonly used for several malignancies including OSCC. On the other hands, the development of intrinsic or acquired cisplatin resistance in OSCC chemotherapy is a major obstacle to its successful clinical application in OSCC. Although there are reports that the involvement of transporters and suppression of apoptosis contribute to resistance in many types of cancer [5,6], detailed molecular mechanisms to cisplatin resistance have yet to be determined in OSCC.

The *cylindromatosis* (*CYLD*) gene, a tumor suppressor, has been initially identified as a responsible mutated gene because its loss causes a benign human tumor called cylindromatosis. Subsequent studies have revealed that CYLD serves as a deubiquitinating enzyme and negatively regulates multiple cell signaling pathways, including nuclear factor-κB (NF-κB), Wnt/β-catenin, c-Jun N-terminal kinase, p38/mitogen-activated protein kinase, and Hippo and Notch signaling [7,8,9,10,11,12,13,14,15,16]. In particular, the NF-κB signaling pathway has been shown to be involved in malignant progression of various types of tumors [17,18]. Previous reports suggested that CYLD served as a negative regulator for NF-κB signaling, and NF-κB overactivation by loss of CYLD function caused the resistance to chemotherapy by suppressing apoptosis [7]. In recent years, reduction of CYLD expression has been detected in various cancers such as breast cancer, glioblastoma, and hepatocellular carcinoma [19,20,21]. It has been reported that both disease-free survival and breast cancer-specific survival were significantly reduced in breast cancer patients with low CYLD expression [19], and overall survival was significantly reduced in hepatocellular carcinoma patients with low CYLD expression [21], suggesting that loss of CYLD expression may be associated with poor prognosis in various cancers. Interestingly, our recent study showed that in OSCC patients, loss of CYLD expression was significantly associated with the clinical features of deep invasion and poor overall survival [22]. Moreover, recent clinical studies have also suggested that the increased NF-κB activity in OSCC tissues may be associated with poor prognosis via chemotherapeutic resistance in OSCC patient [23,24,25,26,27]. However, the detailed mechanisms that lead to a poor prognosis due to loss of CYLD expression in OSCC patients remain largely unknown.

In this study, we focused on cisplatin resistance, one of critical poor prognostic factors in OSCC patients, and determined the relationship between loss of CYLD expression and cisplatin resistance in OSCC cells.

## 2. Results

### 2.1. Effect of CYLD Down-Regulation on Cisplatin Sensitivity in OSCC Cells

**Figure 1:**
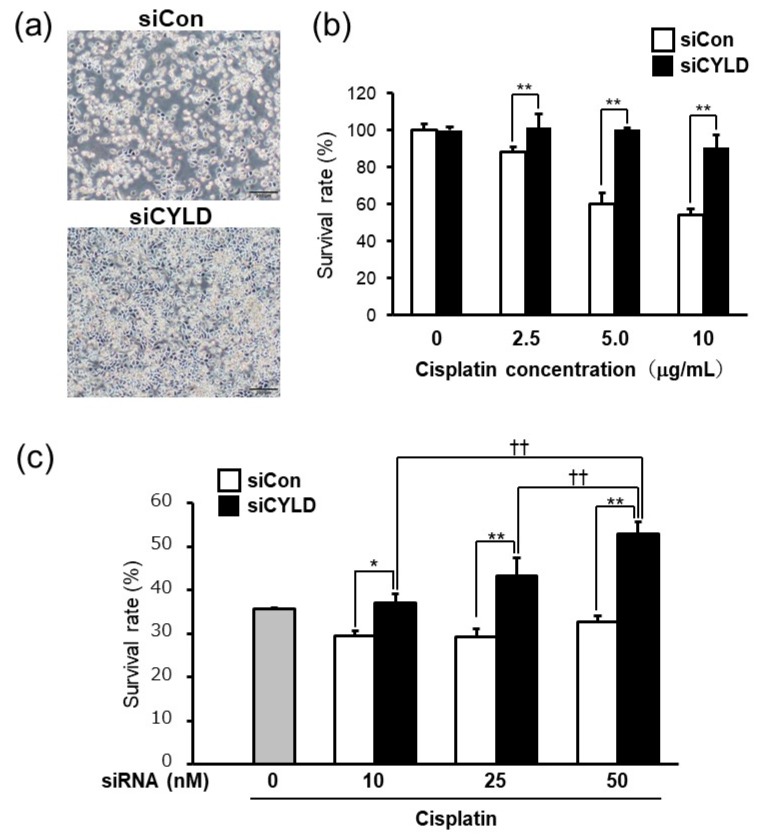
Effect of cylindromatosis (CYLD) down-regulation on cisplatin sensitivity in oral squamous cell carcinoma (OSCC cells). (**a**) Human OSCC cell line (SAS) cells were transfected with control siRNA (siCon) or CYLD-specific siRNA (siCYLD) and then treated with cisplatin (8.0 µg/mL). Scale bar: 200 μm. (**b**) The cell survival rates of SAS cells after cisplatin treatment (0–10 µg/mL) were assessed by MTS assays. (**c**) SAS cells were transfected with 0–50 nM CYLD-specific siRNA. The cell survival rates of SAS cells were evaluated 24 h after cisplatin treatment (8.0 µg/mL). Values are means ± SD of triplicate samples. * *p* < 0.05 and ** *p* < 0.01 vs siCon group. †† *p* < 0.01 vs cisplatin treated siCYLD group.

We first sought to investigate whether CYLD expression affects cisplatin sensitivity in OSCC cells. To determine whether CYLD expression is involved in cisplatin sensitivity in OSCC, we evaluated the effect of CYLD knockdown by CYLD-specific siRNA on cisplatin sensitivity of human OSCC cell line (SAS) cells. As shown in Figure 1a, CYLD-knockdown SAS cells exhibited higher cell viability after cisplatin treatment than did cells transfected with control siRNA. Cell survival rates in CYLD knockdown SAS cells were significantly increased at any concentration of cisplatin treatment (Figure 1b). Moreover, in association with CYLD expression level effects by increasing CYLD-specific siRNA, the cell survival rates in CYLD knockdown SAS cells were significantly increased (Figure 1c), suggesting that CYLD down-regulation caused cisplatin resistance to SAS cells.

### 2.2. CYLD Down-Regulation Induced Cisplatin Resistance through NF-κB Hyperactivation

**Figure 2:**
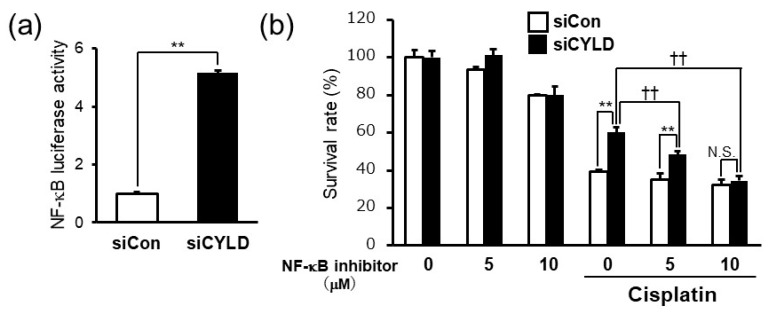
CYLD down-regulation induced cisplatin resistance through NF-κB hyperactivation. (**a**) NF-κB activity was assessed by luciferase reporter assay 48 h after transfection with CYLD-specific siRNA. (**b**) Cell survival rates of SAS cells against cisplatin treatment (8.0 µg/mL) with or without NF-κB inhibitor (BAY 11-7085) were assessed. Values are means ± SD of triplicate samples. ** *p* < 0.01 vs siCon group. †† *p* < 0.01 vs cisplatin treated siCYLD group. N.S.: not significant.

It was reported that CYLD, a deubiquitinating enzyme, negatively regulates the NF-κB signaling pathway in the process of malignant progression [7,8,9,10,11,17,18]. NF-κB activation is known to be involved in the development of resistance to anticancer drugs such as cisplatin and 5-FU in OSCC [26,27]. Since previous clinical studies also showed the NF-κB hyperactivation in OSCC tissues [23,24,25], we next sought to determine whether NF-κB hyperactivation is involved in cisplatin resistance induced by CYLD down-regulation in SAS cells. Consistent with the previous reports about CYLD function as a negative regulator of NF-κB signaling pathway, CYLD knockdown by CYLD-specific siRNA significantly enhanced the NF-κB activity in SAS cells (Figure 2a). Moreover, BAY 11-7085, an irreversible NF-κB inhibitor, significantly suppressed cisplatin resistance induced by CYLD knockdown in a dose-dependent manner (Figure 2b). These results suggested that CYLD down-regulation induced cisplatin resistance through NF-κB hyperactivation in OSCC cells.

### 2.3. Effect of CYLD Down-Regulation on Intracellular Cisplatin Accumulation and Cisplatin-Induced Apoptosis in OSCC Cells

**Figure 3:**
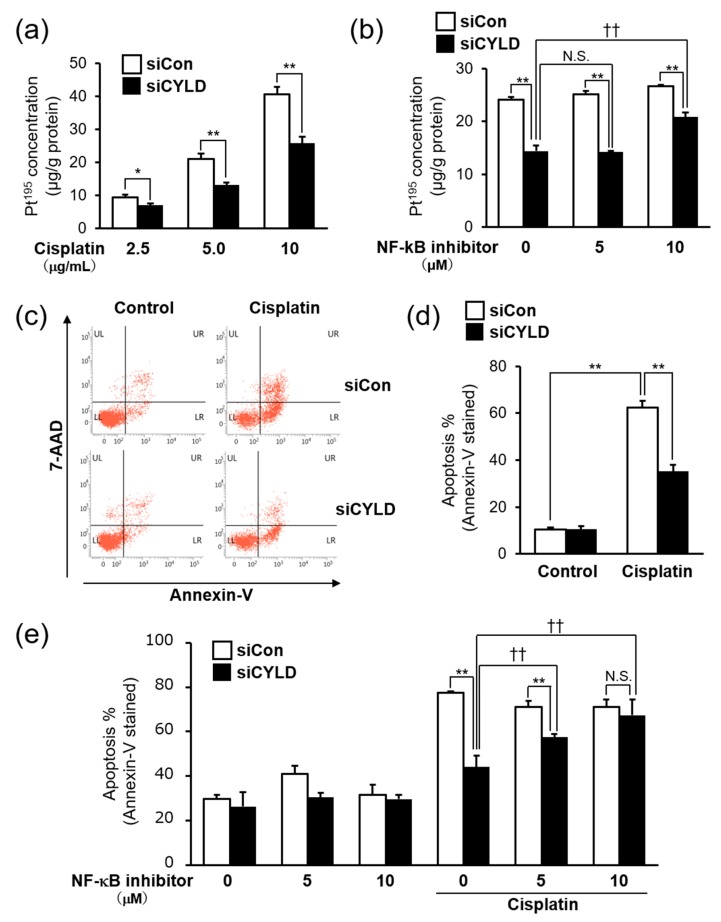
Effect of CYLD down-regulation on intracellular cisplatin accumulation and cisplatin-induced apoptosis in OSCC cells. (**a**,**b**) Measurement of intracellular cisplatin accumulation in SAS cells using ICP-MS. Cells were treated with various cisplatin concentrations (0–10 µg/mL) for 6 h (**a**) and pretreated with or without NF-κB inhibitor (BAY 11-7085, 0–10 μM) 1 h before cisplatin treatment (**b**). (**c**–**e**) The cisplatin-induced apoptosis was assessed by Annexin-V/7-AAD staining using flow cytometry. Cells were pretreated with or without 10–15 μM NF-κB inhibitor (BAY 11-7085) 1 h before cisplatin treatment (8.0 µg/mL) (**e**). Values are means ± SD of triplicate samples. * *p* < 0.05 and ** *p* < 0.01 vs. siCon group. †† *p* < 0.01 vs cisplatin treated siCYLD group. N.S.: not significant.

One of the main factors for cisplatin resistance has been the reduction of intracellular cisplatin accumulation [28,29,30]. We thus tried to determine the level of cisplatin accumulation in CYLD-knockdown SAS cells by using ICP-MS. As shown in Figure 3a, the intracellular cisplatin accumulation was significantly reduced by CYLD knockdown at any cisplatin concentration. In addition, the reduced intracellular cisplatin accumulation in CYLD-knockdown SAS cells was recovered, at least in part, by NF-κB inhibitor (BAY 11-7085) treatment (Figure 3b). Those results suggested that the reduction of intracellular cisplatin accumulation caused by NF-κB hyperactivation was partially involved in cisplatin resistance caused by CYLD down-regulation in SAS cells.

It is well-documented that DNA damage and subsequent induction of apoptosis may be the primary cytotoxic mechanism of cisplatin [31,32]. In contrast, it has been also reported that one of the mechanisms of cisplatin resistance is suppression of cisplatin-induced apoptosis in various types of malignant tumors [28,29]. Since previous studies showed that loss of CYLD function leads to the suppression of apoptosis by NF-κB hyperactivation in malignant tumors [7,8,9,10,11], we next sought to determine whether the suppression of apoptosis was involved in cisplatin resistance caused by CYLD down-regulation in SAS cells. The percentage of apoptotic SAS cells after cisplatin treatment was evaluated by Annexin-V/7-AAD staining using flow cytometry analysis. As shown in Figure 3c,d, the percentage of apoptotic cells treated with 8.0 μg/mL cisplatin was remarkably reduced in CYLD-knockdown SAS cells. Furthermore, to investigate whether the NF-κB hyperactivation caused by CYLD down-regulation was involved in the suppression of cisplatin-induced apoptosis, we also examined the effect of NF-κB inhibitor (BAY 11-7085) treatment. As expected, the suppression of cisplatin-induced apoptotic SAS cells caused by CYLD knockdown was completely restored by NF-κB inhibitor treatment in a dose-dependent manner (Figure 3d). Thus, those results suggested that cisplatin resistance caused by CYLD down-regulation was associated with the mechanism through which both the reduction of intracellular cisplatin accumulation and the suppression of cisplatin-induced apoptosis, via the NF-κB hyperactivation, occurred.

### 2.4. Bortezomib, a Proteasome Inhibitor, Released the Cisplatin Resistance Caused by CYLD Down-Regulation in OSCC Cells

The results shown in Figure 1, Figure 2 and Figure 3 indicated that the NF-κB hyperactivation played important roles in cisplatin resistance caused by CYLD down-regulation via the reduction of intracellular cisplatin accumulation and the suppression of cisplatin-induced apoptosis in SAS cells. Those findings suggested that suppressing NF-κB hyperactivation might be an effective therapeutic strategy for cisplatin resistant OSCC patients with loss of CYLD expression. Bortezomib, a specific proteasome inhibitor, suppresses the NF-κB activation by inhibiting proteasome-mediated degradation of IκB [33]. Since bortezomib was approved and clinically available for treatment of several malignant tumors, such as multiple myeloma and mantle cell lymphoma [34], we verified the therapeutic effectiveness of bortezomib treatment for cisplatin resistance caused by CYLD down-regulation in SAS cells.

**Figure 4:**
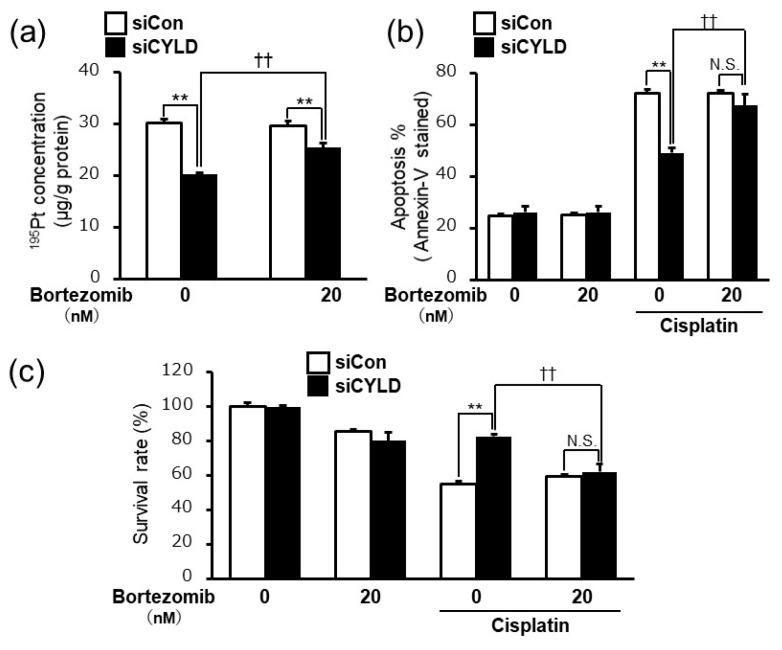
Bortezomib, a proteasome inhibitor, released cisplatin resistance caused by CYLD down-regulation in OSCC cells. (**a**) Measurement of intracellular cisplatin accumulation in SAS cells by using ICP-MS. SAS cells were pretreated with or without bortezomib (20 nM) 1 h before cisplatin treatment (8.0 µg/mL). (**b**) Measurement of cisplatin-induced apoptosis was assayed by Annexin-V/7-AAD staining using flow cytometry. SAS cells were pretreated with or without bortezomib (20 nM) 1 h before cisplatin treatment (8.0 µg/mL). (**c**) The cell survival rates of SAS cells with or without bortezomib (20 nM) after cisplatin treatment (10 µg/mL) were assessed. Values are means ± SD of triplicate samples. ** *p* < 0.01 vs siCon group. †† *p* < 0.01 vs cisplatin treated siCYLD group. N.S.: not significant.

As shown in Figure 4a,b, bortezomib treatment significantly inhibited both the reduction of intracellular cisplatin accumulation and the suppression of cisplatin-induced apoptosis in CYLD-knockdown SAS cells. Furthermore, as expected, the combination of cisplatin and bortezomib treatment exhibited significant anti-tumor effects on cisplatin resistance caused by CYLD down-regulation in SAS cells (Figure 4c). Taken together, our results indicated that bortezomib treatment may serve as a potential therapeutic anti-tumor agent against cisplatin resistant OSCC patients with loss of CYLD expression.

## 3. Discussion

Despite advances in early detection, diagnosis, and multimodal treatment, the 5-year survival rate in OSCC has not improved dramatically over the past 30 years [3]. It is noted that even after remission with OSCC patients by surgical operation, disorders of oral function, such as food intake, swallowing, and speech, still remain because of local characteristics of OSCC. Therefore, development of minimally invasive non-surgical treatments based on the individual status of OSCC patients, especially a novel anti-cancer drug treatment, is urgently required. Since cisplatin resistance is known to be a major obstacle to its successful clinical application in OSCC, causing recurrence, metastasis, and leading to a poor prognosis [4], understanding the mechanism underlying cisplatin resistance is thought to lead to improvements in the mortality of OSCC patients. 

Our results revealed that the loss of tumor suppressor gene CYLD expression caused cisplatin resistance in SAS cells. The cisplatin resistance caused by CYLD down-regulation was also confirmed in other OSCC cells (HSC-3 and Ca9-22) (Supplemental Figure S1). We previously reported that the decreased CYLD expression in OSCC tissue contributed to poor survival prognosis [22]. In this report, immunohistochemical analyses showed significantly reduced CYLD expression in invasive areas in OSCC tissues. Lower expression of CYLD was associated with the clinical features of deep invasion and poor overall survival in OSCC patients. Moreover, down-regulation of CYLD promoted the cell invasion with mesenchymal transition in OSCC cells. Therefore, our finding indicates the possibility that cisplatin resistance caused by CYLD down-regulation is one of the factors of poor prognosis in OSCC patients with low-CYLD expression. Indeed, although the number of clinical cases is still limited, we obtained preliminary evidence to determine the effect of CYLD expression on the therapeutic effects of cisplatin in OSCC patients (data not shown). Superselective intra-arterial chemoradiotherapy, a definitive therapy for patients with advanced cancer, is known to be highly effective as an organ and function preserving therapy [35]. For the patients who were first treated with this chemotherapy using cisplatin, the survival rate during the observation period was as high as 79.4% (27/34 cases). Among those, the OSCC patients with low-CYLD expression showed a tendency to exhibit low therapeutic effect by this chemoradiotherapy (4/7 cases). In addition, no immunohistochemical staining for CYLD was detected in the tissue of these patients with recurrence or metastasis during cisplatin treatment (data not shown). Further investigation by accumulating the clinical evidence will be definitely required to verify the relationship between CYLD expression and therapeutic effects of anti-cancer drugs.

It has been shown that CYLD regulates various cell signaling pathways as a deubiquitinating enzyme [7,8,9,10,11,12,13,14,15,16]. In several types of tumors, CYLD negatively regulates the NF-κB signaling pathway, an important signaling in malignant progression [7,8,9,10,11,17,18]. The results, as shown in Figure 2, suggested that NF-κB hyperactivation caused by CYLD down-regulation played important roles in cisplatin resistance in OSCC patients with low CYLD expression. Although previous reports suggested that the NF-κB activation might contribute to the development of resistance to anti-cancer drugs such as cisplatin and 5-FU in OSCC [26,27], the molecular mechanism for NF-κB pathway activation in OSCC patients has largely yet to be determined. Thus, this is the first report suggesting that NF-κB hyperactivation is caused by down-regulation of CYLD expression in OSCC cells. Previous reports have suggested that CYLD may regulate various steps of the NF-κB signaling pathway by deubiquitinating signaling molecules, such as TNF receptor-associated factors 2 or 6, NF-κB essential modulator, and TGF-β-activated kinase 1 [7,8,9]. Future studies will focus on identifying the molecular target of CYLD to regulate the NF-κB signaling pathway, and the factors triggering the down-regulation of CYLD expression in OSCC cells. The fact that CYLD may be involved in cisplatin sensitivity in OSCC through NF-κB hyperactivation has the potential to contribute to the development of novel therapeutic strategies and improvement of the prognosis of OSCC.

Another finding worth noting is that cisplatin resistance caused by CYLD down-regulation was associated with the mechanism through which both the reduction of intracellular cisplatin accumulation and the suppression of cisplatin-induced apoptosis, via the NF-κB hyperactivation, occurred (Figure 3). One of the cisplatin resistance mechanisms is reduced drug accumulation [28,29,30]. Indeed, in CYLD knockdown cells, cisplatin accumulation was significantly reduced (Figure 3a). Additionally, the reduced intracellular cisplatin accumulation in CYLD-knockdown SAS cells was recovered, at least in part, by NF-κB inhibitor (BAY 11-7085) treatment (Figure 3b). Moreover, in preliminary experiments, we also found that the excretion of cisplatin from SAS cells was increased by CYLD down-regulation (data not shown). It was reported that in cisplatin resistant cells, the expression of efflux transporter (MDR1, MRP1, etc.) of cisplatin is up-regulated [36,37]. It has also been reported that MDR1 expression is regulated by the NF-κB signaling pathway [38]. Therefore, to elucidate the molecular mechanism how cisplatin accumulation is reduced by CYLD down-regulation, further investigation will be needed to identify the efflux transporter of cisplatin regulated by CYLD. On the other hands, it is also known that cisplatin causes cell death by inducing apoptosis [31,32], and one of the mechanisms of cisplatin resistance has been reported to be the suppression of cisplatin-induced apoptosis [28,29]. As shown in Figure 3c,d, the percentage of apoptotic cells by cisplatin treatment was remarkably reduced in CYLD-knockdown SAS cells. Furthermore, the suppression of cisplatin-induced apoptotic SAS cells caused by CYLD knockdown was completely restored by NF-κB inhibitor treatment (Figure 3d). It was documented that the NF-κB signaling pathway regulates the expression of anti-apoptotic proteins, such as Bcl-2, Bcl-xL, and XIAP, and controls the induction of apoptosis by suppressing the release of cytochrome c from the mitochondria to the cytoplasm, thereby promoting survival [36,39]. Previous studies suggested that in cisplatin sensitive cells, the expression of these anti-apoptotic proteins was reduced by cisplatin treatment, while this reduction was not observed in cisplatin resistant cells [40,41,42]. In our preliminary experiments, the expression of anti-apoptotic protein was not reduced by cisplatin treatment in CYLD knockdown cells (data not shown). However, we have not obtained enough evidence on how NF-κB hyperactivation by CYLD knockdown suppressed cisplatin-induced apoptosis. Further studies will focus on elucidating the relationship between CYLD expression and anti-apoptotic status through NF-κB hyperactivation. In addition to these lines of evidence, it was reported that glutathione *S*-transferase-π (GST-π) is responsible for detoxifying xenobiotics and the elevated levels of this enzyme cause treatment resistance [29]. Topoisomerase II is also known to be linked to repair of cisplatin-induced DNA crosslinks, and its overexpression is associated with the onset of cisplatin resistance [29]. Moreover, extracellular pH in solid tumors is more acidic than normal tissues, and it may cause the drug resistance [43]. Recent studies have also suggested that V-ATPases, which secrete protons through the plasma membrane, may play a key role in the acidification of the tumor environment [43]. Thus, in view of these multiple factors, we will further elucidate the mechanisms of cisplatin resistance by CYLD downregulation.

Finally, our results also indicated that the combination of cisplatin and bortezomib significantly inhibited both the reduction of intracellular cisplatin accumulation and suppression of cisplatin-induced apoptosis in CYLD-knockdown SAS cells, and resulted in the suppression of resistance to cisplatin (Figure 4). Bortezomib, a specific proteasome inhibitor, suppresses NF-κB activation by inhibiting proteasome-mediated degradation of IκB [33]. Bortezomib was initially reported as an inhibitor of the NF-κB signaling pathway, which plays critical roles in the pathogenesis of multiple myeloma [34]. As of this moment, bortezomib is clinically available for treatment of several malignant tumors, such as multiple myeloma and mantle cell lymphoma [34]. The results shown in Figure 4a,b indicated that the bortezomib treatment inhibited both the reduction of intracellular cisplatin accumulation and the suppression of cisplatin-induced apoptosis in CYLD-knockdown SAS cells. More importantly, the combination of cisplatin and bortezomib treatment exhibited significant anti-tumor effects on cisplatin resistance caused by CYLD down-regulation in SAS cells (Figure 4c). While bortezomib has been shown to have therapeutic effects in cell lines and mouse xenograft models of OSCC, clinical trials in patients with head and neck squamous cancer carcinoma including OSCC have not yielded the results expected [44,45]. This fact may suggest that if CYLD expression in OSCC tissue can be used as a predictive marker, the combination of cisplatin and bortezomib could be a novel therapeutic strategy for improving the prognosis of OSCC patients with low-CYLD expression. Since bortezomib has not been approved for treatment of OSCC, from the viewpoint of drug-repositioning and early clinical applications, the molecular mechanism underlying cisplatin resistance caused by CYLD down-regulation and the usefulness of CYLD expression as a predictive marker should be further investigated.

In conclusion, the present study revealed that CYLD down-regulation caused cisplatin resistance to SAS cells. This cisplatin resistance caused by CYLD down-regulation was associated with the mechanism through which both the reduction of intracellular cisplatin accumulation and the suppression of cisplatin-induced apoptosis, via the NF-κB hyperactivation, occurred. The bortezomib treatment may serve as a potential therapeutic anti-tumor agent against cisplatin resistant OSCC patients with loss of CYLD expression. Further elucidating the detailed molecular mechanism underlying cisplatin resistance by focusing on CYLD will contribute to identifying the factors that deteriorate the survival prognosis of OSCC and may lead to new therapeutic intervention for OSCC patients.

## 4. Materials and Methods

### 4.1. Reagents

Cisplatin was purchased from Nippon Kayaku (Tokyo, Japan). NF-κB inhibitor (BAY 11-7085, irreversible inhibitor of TNF-α-induced IκBα phosphorylation, #ab141574) was purchased from Abcam (Cambridge, MA, USA). Bortezomib (PS-341, #S1013) was purchased from Selleck (Houston, TX, USA).

### 4.2. Cell Lines and Cell Cultures

The human OSCC cell lines SAS (TKG 0470, Cell Resource Center for Biomedical Research, Cell Bank, Tohoku University) were kindly provided by Dr Yoshida (Department of Oral & Maxillofacial Surgery, Faculty of Life Sciences, Kumamoto University, Kumamoto, Japan). SAS cells were grown in RPMI 1640 (Thermo Fisher Scientific, Inc., Waltham, MA, USA) with 10% heat-inactivated fetal bovine serum (Thermo Fisher Scientific, Inc.) in 5% CO_2_ at 37 °C.

### 4.3. Transfection with siRNA

SAS cells were incubated in 12-well plates (0.8 × 10^5^ cells/mL) for 24 h and were transiently transfected with siRNA (10–50 nM) by using Lipofectamine 2000 (Thermo Fisher Scientific, Inc.) according to the manufacturer’s protocol. After transfection and incubation for 48 h, experiments were performed. Silencer Negative Control siRNA (Thermo Fisher Scientific, Inc.) was used as the control (siCon). The *CYLD* gene siRNA sequences were sense: 5′-GAUUGUUACUUCUAUCAAAtt-3′ and antisense: 5′-UUUGAUAGAAGUAACAAUCtt-3′ (Thermo Fisher Scientific, Inc.).

### 4.4. Measurement of Cell Survival Rate

Cells were incubated in 12-well plates and were treated with different cisplatin concentrations (0–10 µg/mL) in serum-free RPMI 1640. Following exposure, the medium was removed from the wells, the cells were washed with cold 1× PBS (Thermo Fisher Scientific, Inc.) twice, and cells were stained by Trypan blue staining. MTS assay was performed using CellTiter96^®^ AQueous Non-Radioactive Cell Proliferation Assay (Promega, Madison, WI, USA).

### 4.5. NF-κB Reporter Assay

Cell transfected with siRNA (control siRNA or CYLD-specific siRNA) were sequentially co-transfected with NF-κB reporter plasmid (pGL4b vector; Promega, Madison, WI, USA). After 48 h, samples were harvested. Luciferase activities were determined with the Dual-Luciferase Reporter Assay System (Promega).

### 4.6. Inductively Coupled Plasma Mass Spectrometry (ICP-MS)

Cells were incubated in 12-well plates and treated with cisplatin (2.5–10 µg/mL) in serum-free medium. After 3–9 h of incubation, samples were harvested and ICP-MS (Thermo Fisher Scientific, Inc.) was performed. After incubation for different time periods, cells were washed four times with ice-cold PBS and solubilized by direct addition of 1 mL of 70% nitric acid. After 10 min of incubation at room temperature, digested samples were harvested, diluted to 5 mL by addition of 0.5% nitric acid, and analyzed via ICP-MS. All samples were quantitated by using external standard solutions made up in 0.5% nitric acid. The blank solution was 0.5% nitric acid. The protein concentration was measured by using the BCA Protein Assay Kit (Pierce, Rockford, IL, USA) according to the manufacturer’s protocol.

### 4.7. Apoptosis

To assess apoptosis of SAS cells, we used the PE Annexin V Apoptosis Detection Kit I (BD Biosciences, Tokyo, Japan), according to the manufacturer’s protocol, and performed the analysis with the FACS Verse Flow Cytometer (BD Biosciences).

### 4.8. Statistical Analysis

Student’s *t*-test and analysis of variance were used to evaluate differences between the two groups. *p*-values of <0.05 were said to be statistically significant. All data are represented as the mean ± standard deviation (SD).

## Supplementary Materials

The following are available online at https://www.mdpi.com/1422-0067/20/20/5194/s1.

## Abbreviations

CYLD	Cylindromatosis
OSCC	Oral squamous cell carcinoma
NF-κB	Nuclear factor-κB
ICP-MS	Inductively coupled plasma mass spectrometry
siRNA	Small interfering RNA
7-AAD	7-amino-actinomycin D
