# Supplementary material for: Loss of Tumor Suppressor CYLD Expression Triggers Cisplatin Resistance in Oral Squamous Cell Carcinoma
Source: Int J Mol Sci. 2019 Oct 20;20(20):5194. doi: 10.3390/ijms20205194 (PMC6829433; doi:10.3390/ijms20205194)
Supplement: Supplementary file 1 [file ijms-20-05194-s001.pdf]

International Journal of
Molecular Sciences

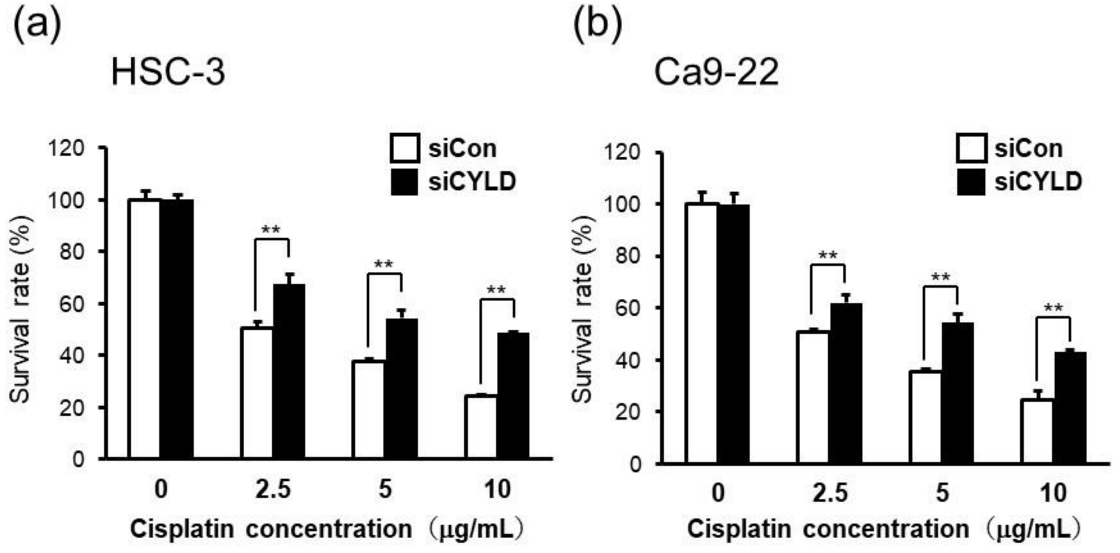

Supplemental Figure 1. Effect of CYLD down-regulation on cisplatin sensitivity in various types of OSCC cell lines. (**a**) HSC-3 cells and (**b**) Ca9-22 cells were transfected with control siRNA (siCon) or CYLD-specific siRNA (siCYLD) and then treated with cisplatin. The cell survival rates of those cells after cisplatin treatment (0 ~ 10 μg/mL) were assessed. Values are means ± SD of triplicate samples. **$p < 0.01$ vs siCon group.

Int. J. Mol. Sci. **2019**, *20*, 5194; doi:10.3390/ijms20205194 www.mdpi.com/journal/ijms
